# Successfully repaired adult cor triatriatum with mitral regurgitation and atrial fibrillation: a case report

**DOI:** 10.1186/s44215-025-00206-5

**Published:** 2025-04-07

**Authors:** Shuhei Naito, Yoshiharu Enomoto, Sei Morizumi, Yuichiro Kaminishi, Bryan J. Mathis, Yasuyuki Suzuki

**Affiliations:** 1 Department of Cardiovascular Surgery, Ibaraki Prefectural Central Hospital, 6528 Koibuchi, Kasama, Ibaraki Japan; 2 Department of Cardiovascular Surgery, Institute of Medicine, University of Tsukuba, 1-1-1 Tennodai, Tsukuba, Ibaraki 305-8575 Japan

**Keywords:** Cor triatriatum, Atrial fibrillation, Mitral regurgitation, Maze procedure

## Abstract

**Background:**

Rarely seen in adults, cor triatriatum is a congenital defect in which a membrane creates three atrial chambers in the heart. Atrial fibrillation (AF) is the most common complication, but repair procedures in adults remain unestablished.

**Case presentation:**

A 49-year-old woman had cor triatriatum sinister (Lucas-Schmidt classification type I-A) with moderate mitral regurgitation and atrial fibrillation. Communication between the accessory chamber and the main chamber was established via a 10-mm fenestration with a pressure gradient of 14 mmHg. Mitral valve repair and the maze procedure, via radiofrequency ablation, was done after complete resection of the anomalous septum in the left atrium. Mitral regurgitation was well-controlled and atrial fibrillation disappeared. Sinus rhythm has been continually maintained at 6 months post-surgery, with no mitral regurgitation recurrence.

**Conclusions:**

Here, a rare adult cor triatriatum case with simultaneous mitral valve regurgitation and atrial fibrillation was treated with good results. The maze procedure shows effectiveness for atrial fibrillation associated with cor triatriatum. Especially the anomalous membrane attachments must be resolved in addition to the conventional maze procedure because electrical mapping of Cor triatriatum sinister showed low-voltage zones at the membrane attachments and it cause macro-reentrant atrial tachycardia.

## Background

Cor triatriatum, a congenital heart defect with an incidence of 0.1% to 0.4% of all congenital cardiac diseases [1], is characterized by a fibromuscular membrane dividing the left (cor triatriatum sinister, CTS) or right atrium (cor triatriatum dexter). In CTS, the left atrium is divided into the posterosuperior chamber (the accessory chamber), which receives blood from pulmonary veins, and the anteroinferior chamber (the main chamber), which contains the left atrium appendage and mitral annulus.

CTS usually presents signs of pulmonary hypertension and pulmonary venous obstruction in childhood but occasionally remains asymptomatic until adulthood. In adult cases, atrial fibrillation (AF, 32.8%) and mitral regurgitation (MR, 24.6%) are common complications [2]. However, reports of adult CTS with both complications are scarce and there is no established maze procedure for AF associated with CTS.

## Case presentation

A 49-year-old woman with no significant previous medical history was referred to our institution due to abnormal findings consisting of AF and an enlarged cardiac shadow on chest X-ray. No such abnormalities were noted during her childhood medical checkups.

**Fig. 1:**
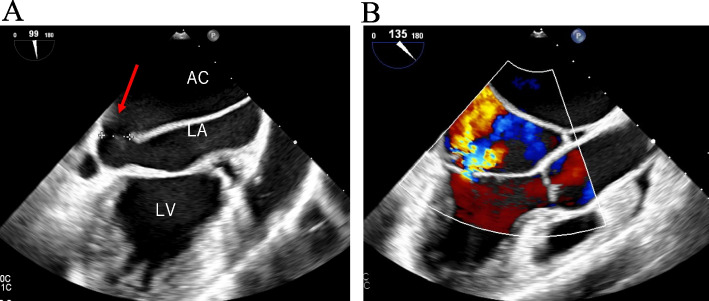
Preoperative transesophageal echocardiography. **A** The left atrium was divided into the accessory chamber and the main chamber by the anomalous membrane (red arrow). **B** The diameter of the fenestration was 11 mm with a peak gradient of 20 mmHg (blue arrow). **C** Moderate mitral regurgitation associated with mitral annual enlargement was found. AC, accessory chamber; MC, main chamber

**Fig. 2:**
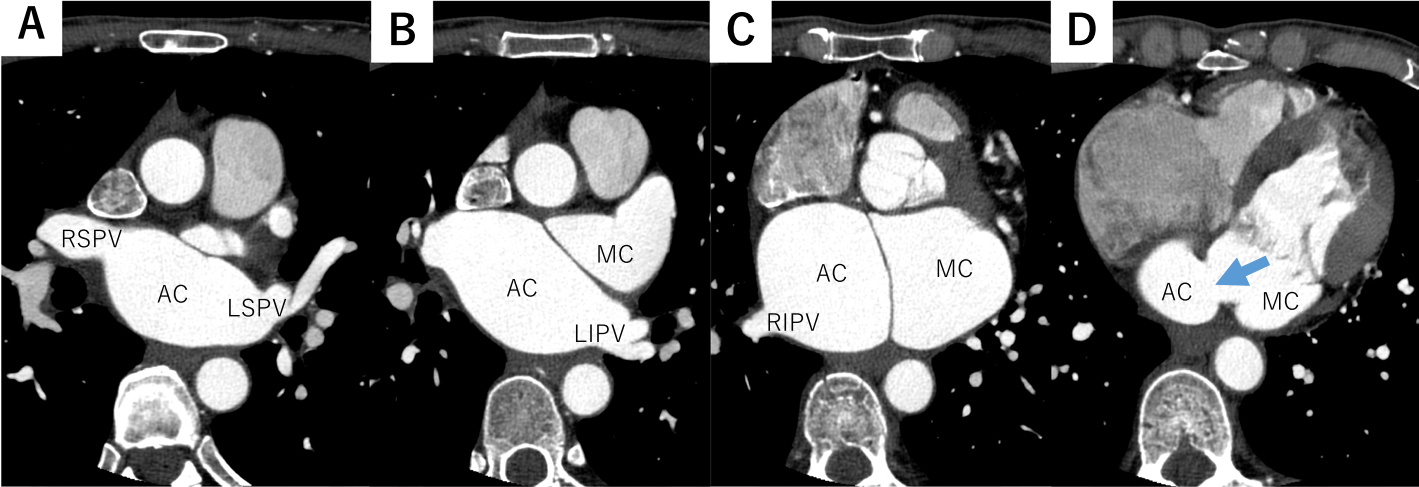
Preoperative enhanced computed tomography. **A–C** All four pulmonary veins entered the heart through the accessory chamber. **D** The accessory chamber and the main chamber communicated via an 11-mm hole (blue arrow). RSPV, right superior pulmonary vein; RIPV, right inferior pulmonary vein; LSPV, left superior pulmonary vein; LIPV, left inferior pulmonary vein; AC, accessory chamber; MC, main chamber

Laboratory tests revealed a raised N-terminal pro-B-type natriuretic peptide of 638 ng/L. Holter electrocardiography showed persistent AF with no sinus rhythm. Transthoracic echocardiography (TTE) showed normal left ventricular function, moderate MR, a severely dilated left atrium, and an anomalous membrane in the left atrium. Tricuspid regurgitation was trivial and tricuspid regurgitation pressure gradient was 31 mmHg. Transesophageal echocardiography (TEE) revealed the presence of the accessory chamber which was separated from the left atrium by the anomalous membrane, leading to a diagnosis of CTS (Fig. 1). There was a communication between the accessory and main chambers through an 11-mm fenestration, with a mean pressure gradient of 9 mmHg. Mitral valves did not appear to be prolapsed and the main cause of MR was thought to be a remarkable dilatation of the annulus. Enhanced computed tomography showed that the accessory chamber received drainage from all pulmonary veins and no other cardiac malformations besides CTS were noted (Fig. 2). On bilateral heart catheterization, the mean main pulmonary artery pressure was 20 mmHg, the mean pulmonary capillary wedge pressure (PCWP) was 24 mmHg, and the mean pressure gradient between PCWP and left ventricular end diastole pressure was 12 mmHg. Based on these findings, the CTS was Lucas-Schmidt classification type I-A. Resection of the anomalous septum, mitral valve repair, left atrial appendage resection, and maze procedure were scheduled.

**Fig. 3:**
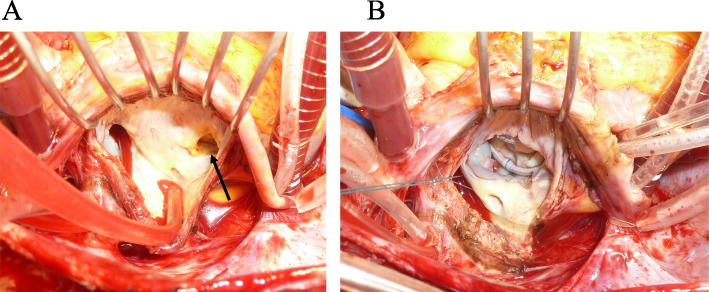
Intraoperative pictures. **A** Anomalous membrane viewed from the accessory chamber side. One fenestration of 11 mm diameter was observed (blue arrow). **B** View after resection of the anomalous membrane and mitral valve repair

**Fig. 4:**
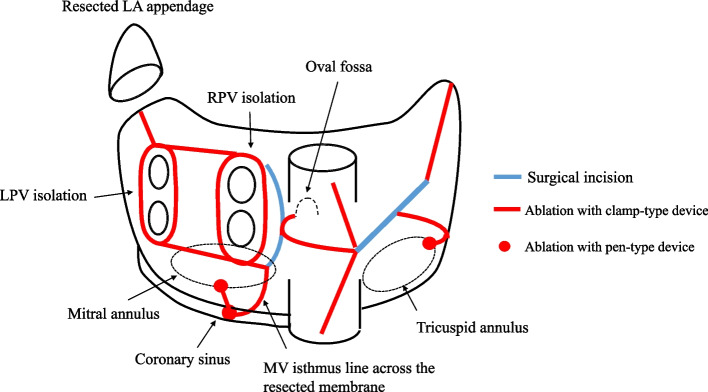
Maze procedure schema. The ablation of MV isthmus line was performed across the remained membrane after resection. LA, left atrium; LPV, left pulmonary veins; RPV, right pulmonary veins; MV, mitral valve

The operation was carried out through a standard median sternotomy. Cardiopulmonary bypass was established with cannulation of the ascending aorta and superior and inferior vena cava. The accessory atrial chamber was incised in front of the right pulmonary veins and the anomalous membrane was recognized (Fig. 3). The fenestration in the anomalous membrane that made the communication between the accessory and main chambers had a diameter of about 12 mm. The anomalous membrane was excised to the fullest extent possible before the maze procedure was completed by AtriCure radiofrequency system (AtriCure Inc., OH, USA) across the remaining part of the membrane from the pulmonary vein isolation line to the mitral annulus (Fig. 4). The anomalous membrane was attached to the left atrial wall near the base of the hypoplastic P3 segment of the mitral valve. MR was thought to be caused by both hypoplasia of the P3 segment and a dilatation of the annulus. We performed mitral valve repair using edge-to-edge sutures during A3-P3 and, after finding the annulus diameter to be 30 mm, ring annuloplasty with a Profile 3D annuloplasty ring (Medtronic, Dublin, Ireland) was conducted. As mitral annulus enlargement was thought to be one of the causes of MR, we used a 28-mm ring to ensure a smaller annulus. Weaning from cardiopulmonary bypass was uneventful and sinus rhythm was maintained. The total bypass and cross-clamp times were 229 min and 180 min, respectively.

Her postoperative course was uncomplicated and discharge occurred on post-operative day 22. Sinus rhythm was maintained until discharge and postoperative TTE revealed no MR. She had been taking warfarin since the postoperative period but, at 6 months after the surgery, AF has not recurred and warfarin was stopped.

## Discussion

Cor triatriatum is rarely seen in adulthood as most repairs occur in children. If no concomitant cardiac malformations exist, the prognosis mainly depends on the size of the membrane fenestration between the accessory and main chambers. Small fenestrations result in pulmonary hypertension due to pulmonary venous return obstruction, mimicking mitral stenosis, in infancy. However, a large fenestration may remain asymptomatic into adulthood. Niwayama reported a survival time of 16.1 years for a fenestration larger than 3 mm and a poor prognosis of 3.3 months for a fenestration smaller than 3 mm [3]. The reasons for conversion to a symptomatic state in adulthood are thought to be 1 or more of the following factors: the fibrosis and calcification of the membrane orifice (resulting in pulmonary venous obstruction), the development of mitral regurgitation, and development of AF. In our case, the fenestration was 11 mm in diameter, supporting the asymptomatic status of the patient during childhood.

Surgical resection of the anomalous membrane is the defining treatment for CTS and is indicated for adults with symptoms attributable to the obstruction or a substantial gradient across the membrane [4]. The efficacy and safety of surgical resection of the anomalous membrane have been reported in several cases. On the other hand, in cases with no pressure gradient at diagnosis, it was reported that conservative treatment did not increase the pressure gradient [5].

MR is associated with 24.6% of CTS [2]. Causes of MR have been reported to include prolapse and myxomatous degeneration of the leaflet [6], as well as mitral annulus enlargement associated with AF [7], but a direct relationship to CTS is unclear. This case was initially thought to be due to valve ring enlargement from AF but, during the operation, it was found that a hypoplastic mitral valve leaflet was also involved in the MR as it was very close to the membrane attachments. To our knowledge, no similar case has been reported but this case suggests that an abnormal membrane may also affect mitral valve morphology in CTS.

AF is the most common complication of CTS, at 32.8% of reported cases, and incidence increases with age [2]. There are few reports of the maze procedure for AF complicated by CTS [6–8]. The mechanism by which AF appears in CTS is unclear but the anomalous membrane may play an important role. In histological terms, the anomalous membrane contains a myocardial structure with severe fibrotic degeneration, which may contain a micro- or macro-reentry circuit [7]. In electrophysiological terms, it has been reported that, during electrical mapping of CTS, low-voltage zones are found at the membrane attachments and cause macro-reentrant atrial tachycardia [9]. Therefore, the anomalous membrane attachments must be resolved in addition to the conventional maze procedure. There are no extant reports on the recurrence rate of AF under these circumstances but, in case reports, Nakajima and Masding reported no recurrence at 3 years and 6 months, respectively [7, 8].

## Conclusion

We experienced a surgical case of CTS diagnosed in adulthood, complicated by AF and MR. We performed a resection of the anomalous membrane, mitral valve repair, and maze procedure with good results. In AF associated with CTS, reentry is expected on the anomalous membrane and its attachment, so it is important to resect the membrane as much as possible and ablate the atrium myocardium of the attachment.

## Data Availability

Not applicable.

## Abbreviations

CTS: Cor triatriatum sinister
AF: Atrial fibrillation
MR: Mitral regurgitation
TTE: Transthoracic echocardiography
TEE: Transesophageal echocardiography
PCWP: Pulmonary capillary wedge pressure
LVEDP: Left ventricular end diastole pressure
